# Gemcitabine and cisplatin in a multimodality treatment for locally advanced non-small cell lung cancer

**DOI:** 10.1038/sj.bjc.6600044

**Published:** 2002-01-21

**Authors:** C H Yang, C M Tsai, L S Wang, Y C Lee, C J Chang, L T Lui, S H Yen, C Hsu, A L Cheng, M Y Liu, S C Chiang, Y M Chen, K T Luh, M H Huang, P-C Yang, R-P Perng

**Affiliations:** Department of Oncology and Cancer Research Center, National Taiwan University Hospital, College of Medicine, National Taiwan University, 7, Chung-Shan South Road, Taipei, Taiwan 10016; Department of Internal Medicine, National Taiwan University Hospital, College of Medicine, National Taiwan University, 7, Chung-Shan South Road, Taipei, Taiwan 10016; Department of Surgery, National Taiwan University Hospital, College of Medicine, National Taiwan University, 7, Chung-Shan South Road, Taipei, Taiwan 10016; Department of Clinical Medicine, National Taiwan University Hospital, 7, Chung-Shan South Road, Taipei, Taiwan 10016; Department of Radiotherapy, National Taiwan University Hospital, College of Medicine, National Taiwan University, 7, Chung-Shan South Road, Taipei, Taiwan 10016; Chest Department, Taipei Veterans General Hospital, 201, Sec 2, Shih-Pai Road, Taipei, Taiwan 11217; Department of Surgery, Taipei Veterans General Hospital, 201, Sec 2, Shih-Pai Road, Taipei, Taiwan 11217; Cancer Treatment Center, Taipei Veterans General Hospital, 201, Sec 2, Shih-Pai Road, Taipei Veterans General Hospital, Taipei, Taiwan 11217; School of Medicine, National Yang–Ming University, Taiwan

**Keywords:** locally advanced NSCLC, neoadjuvant chemotherapy, gemcitabine

## Abstract

The role of new cytotoxic agents like gemcitabine has not yet been proven in the neoadjuvant settings. We designed a phase II study to test the feasibility of using gemcitabine and cisplatin before local treatment for stage III non-small cell lung cancer patients. Patients received three cycles of induction chemotherapy of gemcitabine (1000 mg m^−2^, days 1, 8, 15) and cisplatin (90 mg m^−2^, day 15) every 4 weeks before evaluation for operability. Operable patients underwent radical resection. Inoperable patients and patients who had incomplete resection received concurrent chemoradiotherapy with daily low dose cisplatin. All patients who did not progress after local treatment received three more cycles of adjuvant chemotherapy of gemcitabine and cisplatin. Fifty-two patients received induction treatment. Two patients had complete response and 31 patients had partial response (response rate 63.5%) after induction chemotherapy. Thirty-six patients (69%) were operable. Eighteen patients (35%) had their tumours completely resected. Two patients had pathological complete response. Median overall survival was 19.1 months, projected 1-year survival was 66% and 2-year survival was 34%. Three cycles of gemcitabine and cisplatin is effective and can be used as induction treatment before surgery for locally advanced non-small cell lung cancer patients.

*British Journal of Cancer* (2002) **86**, 190–195. DOI: 10.1038/sj/bjc/6600044 www.bjcancer.com

© 2002 The Cancer Research Campaign

## 

Non-small cell lung cancer (NSCLC) is the leading cause of cancer death in the world. Most patients are diagnosed with inoperable disease at the time of presentation. Novel chemotherapeutic agents have been shown in some studies to increase survival of stage IIIB/IV patients. However, these patients were cured rarely, if ever, by current chemotherapeutic agents (Hoffman *et al*, 2000). On the other hand, several phase III studies have shown that cisplatin-containing induction chemotherapy may increase survival rate in stage IIIA or IIIB locally advanced non-small cell lung cancer (LAD-NSCLC) patients. Two randomized studies demonstrated increased survival in stage IIIA NSCLC patients if two to three cycles of chemotherapy were given before surgery (Rosell *et al*, 1994; Roth *et al*, 1994). In inoperable patients, induction with cisplatin plus vinblastine followed by radiotherapy demonstrated long-term survival advantage over radiotherapy alone in a randomized study (Dillman *et al*, 1996) and was confirmed subsequently by an intergroup study (Sause *et al*, 1995). With the encouraging effects of novel agents in inoperable NSCLC patients, it is conceivable that replacing conventional cisplatin combinations with induction regimens containing new agents may further improve survival in LAD-NSCLC patients.

Gemcitabine plus cisplatin (GC) is one of the most active regimens in the treatment for stage IIIB/IV NSCLC patients. In phase II studies, 26–54% stage IIIB/IV NSCLC patients responded to GC treatment. Good median and 1-year survival have been consistently observed (Abratt *et al*, 1997; Crino *et al*, 1997; Einhorn, 1997; Shepherd *et al*, 1997). Three randomized phase III studies demonstrated the superiority of GC over other chemotherapy regimens (Cardenal *et al*, 1999; Crino *et al*, 1999; Sandler *et al*, 2000). It is likely that GC as an induction treatment may further improve survival in LAD-NSCLC patients. Abratt *et al* (1997) used GC in stage IIIB/IV patients delivering cisplatin on day 15 resulted in good response rate (52%), median survival (13 months) associated with low haematological toxicity and very few dose modifications of either gemcitabine or cisplatin. This schedule seems to be feasible for induction treatment. Based on these observations, we designed a combined modality approach to LAD-NSCLC patients, using the GC regimen with a day 15 cisplatin schedule as was used in the Abratt *et al* (1997) study as induction chemotherapy.

Local recurrence occurs at high frequencies during follow-up in patients with LAD-NSCLC (Le Chevalier *et al*, 1994). In a randomized trial, daily low dose cisplatin (6 mg m^−2^) as radiation sensitizer given concurrently with radiotherapy reduced local recurrence (Schaake-Koning *et al*, 1992). Overall survival was increased in patients who received daily low dose cisplatin (16% *vs* 2%, 3 year survival). Daily low-dose cisplatin was given concurrently with radiotherapy in the present study.

## PATIENTS AND METHODS

### Patients selection

Eligibility criteria included pathologically or cytological proven LAD-NSCLC (stage IIIA/IIIB) (Mountain, 1986) patients. Both inoperable and operable patients were allowed to enter the study. T3, T4 or N2, N3 disease must be documented by any of the following criteria: direct visualization of invasion by tumour of carina or bronchus with bronchoscopic examination, pathological or cytological evidence of N2 or N3 disease, unequivocal radiological evidence in computed tomography of chest (such as direct invasion of tumour to vital organs, bulky and multiple mediastinal lymph nodes). Mediastinoscopy was not a prerequisite if there were multiple metastatic lymph nodes and at least one of them was more than 1.5 cm in diameter in a computed tomography of the chest. Patients who had supraclavicular lymph nodes metastasis, pleural or pericardial effusion were excluded from the study. Patients had to be more than 18 years old and had a World Health Organization (WHO) performance status of 0–2. There must be at least one measurable tumour and patient must not have received any prior cancer treatment. Adequate pulmonary function (FEV1 ⩾2.0 L or FEV1 and FVC ⩾50% predicted value) and normal bone marrow reserve (white blood cell count ⩾4000 mm^−3^, neutrophils ⩾1500 mm^−3^, platelet ⩾100 000 mm^−3^, haemoglobin ⩾10 g dL^−1^) and good liver, renal function were required. Patients with superior sulcus tumour, superior vena cava syndrome, metabolic complications, serious systemic, neurological, psychological disease and second malignancies were excluded. Patients were required to sign informed consent to enter the study.

### Treatment plan

Treatment consisted of intravenous infusion of gemcitabine 1000 mg m^−2^ on days 1, 8, 15 and cisplatin 90 mg m^−2^ on day 15, in a 28-day cycle. After three cycles, patients were restaged and evaluated by computed tomography of the chest for treatment response and operability. Patients were deemed inoperable either if they were T4, N3 or M1 or if they were N2 with multiple levels, bulky size or high mediastinal lymph node involvement. Patients who were operable underwent thoracotomy with curative resection and radical lymph node dissection at least 3 weeks from the last dose of chemotherapy. Concurrent chemoradiotherapy was given to patients who were not candidates for curative surgery or if residual tumour was noted after thoracotomy. Radiotherapy was directed at tumour and lymphatic chains with at least 1 cm margin at 200 cGy/fr, 5 days a week, for a total of 5600 cGy. Low dose cisplatin at 6 mg m^−2^ was given within 30 min before radiotherapy to enhance local control. Three cycles of adjuvant gemcitabine and cisplatin with the same dose and schedule as in induction treatment were given to all patients after local treatment. Treatment was delayed if either haematological or non-haematological treatment-related toxicity greater than grade 2 was observed. Patients must have a leukocytes count of more than 2500 mm^−3^ on day 15 to receive gemcitabine and cisplatin treatment.

### Evaluation of response and toxicity

Patients were evaluated for treatment response using WHO criteria after three cycles of induction chemotherapy, after surgery, after concurrent chemoradiotherapy and at the completion of adjuvant chemotherapy. All scans with documented response were reviewed in regular meetings by investigators of two institutions. Toxicity of the treatment was evaluated using WHO toxicity criteria after every cycle of chemotherapy, after surgery and after concurrent chemoradiotherapy. Patients were followed at least every 3 months after completion of protocol treatment. Computed tomography of the chest was performed every 3 months until documented relapse or progression.

### Statistical considerations

Kaplan-Meier method was used for survival estimates at various time points and their 95% confidence intervals were also presented. Exploratory univariate analyses were performed. Log-rank tests were used to compare overall survival curves in patients with different pre-study factors and treatment related predictive factors. Thirty-eight evaluable patients will provide a less than 2% possibility of failing to observe any severe toxicity if the true rate of severe toxicity exceeded 10%. Thirty-eight evaluable patients will also give a good 95% confidence interval to confirm the 40% response rate after three cycles of gemcitabine plus cisplatin treatment in NSCLC patients. If 25% of accrued patients cannot finish local treatment for various reasons, a total of 51 patients were needed in this study.

## RESULTS

### Patient characteristics

The protocol was approved by Institutional Review Boards. All patients signed informed consent before entering the study. From August 1997 to September 1999, 54 patients were accrued. Two patients were found to have stage IV disease upon review and were excluded. Fifty-two patients were included in this analysis. Patient demographics are listed in Table 1

**Table 1:**
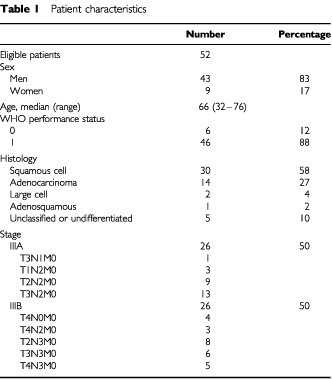
Patient characteristics

. Most patients were men with squamous cell carcinoma histology. Forty-seven patients (90%) had N2 or N3 (cytological or histological proven or unequivocal radiographic evidence of contralateral mediastinum or hilar lymph nodes involvement, patients with supraclavicular or neck lymph nodes metastasis were excluded) disease. Mediastinoscopic biopsy was performed on 17 patients, with positive lymph node involvement. All other patients who had clinically N2 or N3 disease had multiple mediastinal lymph nodes, and at least one of the lymph nodes measuring more than 1.5 cm in size in a computed tomography of the chest. Thoracic surgeons reviewed all of the clinical and radiological findings. Forty-eight patients had unresectable tumours and four patients (one T3N1 and three patients with T2N2) had resectable tumours.

### Induction treatment, local treatment and adjuvant chemotherapy

**Figure 1:**
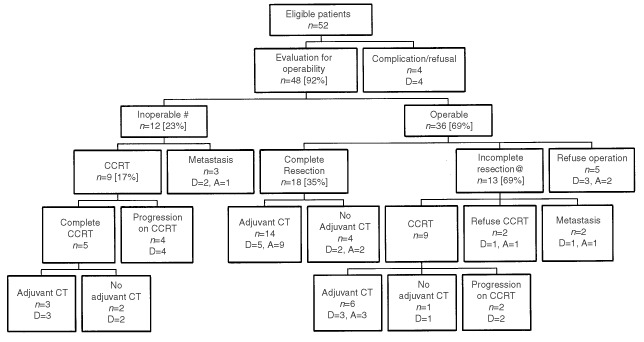
Summary of the treatment and outcome of 52 patients. Abbreviations: N: patient number, D; dead, A; alive, CCRT: concurrent chemoradiotherapy, CT: chemotherapy. #In 12 inoperable patients, three had metastasis after three cycles of neoadjuvant treatment, nine had only locoregional tumours, but the tumours did not meet criteria for operation. These nine patients all received concurrent chemoradiotherapy. @In 13 patients who had incomplete resection, two had metastasis and 11 had unresectable locoregional tumour found during operation.

The treatment and outcome of patients are summarized in Figure 1

. Fifty-two patients completed a total of 148 cycles of induction chemotherapy. Forty-five patients (87%) finished three planned cycles. Seven patients did not complete chemotherapy. Two patients died of complications (one hepatitis, one pneumonia). Two patients refused scheduled chemotherapy. Three patients progressed during neoadjuvant treatment and were withdrawn. Forty-eight patients (92%) were evaluated for operability after induction treatment. Thirty-six patients (69%) were operable and 12 patients (23%) were inoperable after induction chemotherapy.

Among 36 operable patients, five refused surgery, 31 patients underwent thoracotomy and lymph node dissection. Eighteen patients (35%) had their tumours completely resected whereas complete resections were not possible in 13 (25%) patients. Among these 13 patients, two were found to have disseminated disease during surgical exploration and 11 patients had unresectable localized disease. Eighteen patients received concurrent chemoradiotherapy as salvage treatment (nine patients after incomplete resection during surgery plus nine inoperable patients). Only 12 patients completed chemoradiotherapy. Six patients had progressive disease during concurrent chemoradiotherapy.

Among 30 patients who were able to finish local treatment (surgery and/or concurrent chemoradiotherapy), 23 (44%) were able to receive adjuvant chemotherapy. Twenty (38%) of them completed three cycles of treatment.

### Toxicity of treatment

Side effects of induction chemotherapy of 52 patients are listed in Table 2

**Table 2:**
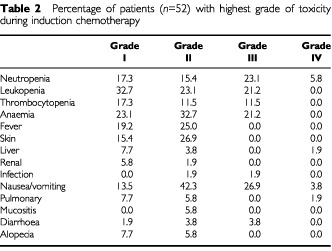
Percentage of patients (*n*=52) with highest grade of toxicity during induction chemotherapy

. Two patients died during induction chemotherapy. One patient died of reactivation of hepatitis B virus. One patient died of pneumonia, probably related to chemotherapy. One patient died of infection after operation. There were no other patients with severe perioperative morbidity after induction chemotherapy. Side effects of concurrent chemoradiotherapy included grade III/IV neutropenia (22/28%), thrombocytopenia (17/28%), mild oesophagitis (44%), mild radiation pneumonitis (17%) and grade III/IV vomiting (6/11%). Side effects of adjuvant chemotherapy were similar to neoadjuvant chemotherapy except that haematological toxicity was higher in adjuvant chemotherapy (grade 3/4 neutropenia, 35 *vs* 29%; thrombocytopenia 35 *vs* 12% and anaemia 43 *vs* 21%).

### Treatment delays

The protocol was designed to delay treatment until resolution of treatment related toxicity instead of modifying the dose of treatment. In 148 treatment cycles, only in 12 cycles (8%) were the treatment delayed for more than 7 days. One hundred and four cycles (70%) were given within 3 days of scheduled dates. For 45 patients who received three cycles of gemcitabine and cisplatin, three cycles of chemotherapy were completed between 83 to 109 days (median 91 days). The median time to local treatment in 40 patients who received either surgery or concurrent chemoradiotherapy was 107 days (range 87 to 169 days). Patients who received three cycles of full dose induction chemotherapy and surgery or concurrent chemoradiotherapy on day 99 (three cycles of 28-day cycle plus 14 days of rest before local treatment) was defined as receiving relative dose intensity of 100%. Median relative dose intensity of induction chemotherapy given for patients was 92.3%.

### Treatment response, survival and failure pattern

Two patients (3.8%) had clinical complete response after neoadjuvant chemotherapy. Thirty-one patients (59.6%) had partial response. Response rate was 63.5% (95% confidence interval 50.4–76.6%). Eleven (21.1%) had stable disease and four had progressive disease (7.7%). Four patients were not evaluable for response (two complications, two refusals). Pathological samples were examined in 31 operated patients. Two patients (4%) had pathological complete response.

**Figure 2:**
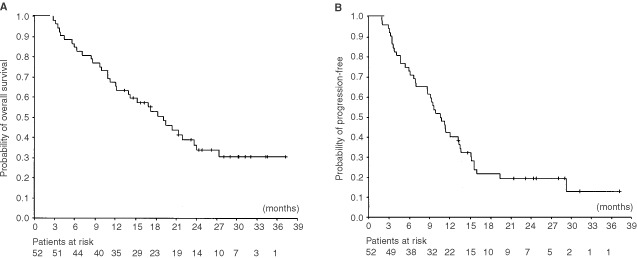
(**A**) Overall survival and (**B**) progression-free survival curves for 52 patients. Patients at risk are given at the bottom.

After a median follow-up time of 26.4 months (range 2.8–37.3 months), 33 of 52 patients were dead. Probability of survival was estimated by Kaplan-Meier method and is shown in Figure 2

. The median overall survival for all patients was 19.1 months (95% confidence interval 14.9–23.3 months), 1-year survival was 66% and 2-year survival was 34%. Median progression-free survival was 10.6 months (95% confidence interval 8.4–12.8 months). The outcomes of all 52 patients are listed in Figure 1. Ten patients (19%) were progression-free at the time of analysis.

In order to find out predictive factors for better survival, subsequent survival analysis were performed by dividing patients into subsets according to different levels of stage, histology, age, operability after induction treatment, nodal status in pathology specimen, response after induction, response after local treatment and time-to-local-treatment. Exploratory Kaplan-Meier estimates based on subsets of patients were derived and overall survival rates within the subsets are compared using log-rank test. Three factors (operable after induction treatment, partial or complete response after local treatment and short time-to-local-treatment) predicted for favourable outcomes.

All patients were followed to detect first relapse or progression site. Patients who did not receive any local treatment were not included in the analysis. The information was available in 32 patients. Twelve patients had locoregional (within surgical or radiation field) progression or relapse, 16 patients had distant relapse, four patients had both locoregional and distant as first relapse sites. Fifteen out of these 32 patients have received adjuvant chemotherapy. Five had locoregional progression, seven had distant relapse and three had both locoregional and distant progression.

## DISCUSSION

In the current study, three cycles of gemcitabine and cisplatin induced response in 63% of patients. The toxicities of induction treatment were acceptable. The major difference in toxicity between this and prior EORTC study (Van Zandwijk *et al*, 2000) was in rates and degrees of thrombocytopenia. There were only 11% of patients with grade 3 thrombocytopenia in this study. Only 8% of the cycles were delayed. On the contrary, when cisplatin was given on day 2, 60% patients had grade 3/4 thrombocytopenia in 46% treatment cycles and the haematological toxicity resulted in day 15 reduction (23%) and omissions (34%) in many cycles. The difference in toxicity was due to the different dose and schedule used in Van Zandwijk *et al* (2000) study (gemcitabine 1000 mg m^−2^ on days 1, 8, 15 and cisplatin 100 mg m^−2^ on day 2). Our study suggests that current schedule (gemcitabine 1000 mg m^−2^ on days 1, 8, 15 and cisplatin 90 mg m^−2^ on day 15) is feasible as induction treatment for LAD-NSCLC patients.

The Southwest Oncology Group reported a mature result of concurrent cisplatin, etoposide with local irradiation as induction therapy in inoperable stage III NSCLC patients. (Albain *et al*, 1995). The response rate was 59%. Eighty-five per cent of patients were operable after induction treatment. Pathological complete remission was documented in 8% of patients. Median and 2-year survivals were 13, 17 months and 37%, 39% for stage IIIA and IIIB patients, respectively. A Cancer and Leukaemia Group B study used a cisplatin, vinblastine and 5-fluorouracil with radiotherapy in 41 stage IIIA NSCLC patients. The response rate after concurrent chemoradiotherapy was 51%. Seventy-four per cent of patients were able to undergo surgery, 59% were completely resected. Complete pathological response was noted in 9% of patients. The median survival was 15.5 months (Strauss *et al*, 1992). The results of the current and the aforementioned EORTC study (Van Zandwijk *et al*, 2000) suggest that GC regimen has achieved similar efficacy to cisplatin-based concurrent chemoradiotherapy as induction treatment in LAD-NSCLC patients. Incorporation of induction chemotherapy with concurrent chemoradiotherapy followed by radical surgery in the intensive tri-modal therapy has been successful in treating LAD-NSCLC patients (Eberhardt *et al*, 1998; Thomas *et al*, 1999). However, similar survival rates can be obtained with bi-modal treatment without surgery (Choy *et al*, 1998; Vokes *et al*, 1999). Thus, the benefit of surgery after intensive chemoradiotherapy remains unclear.

Locoregional tumours, distant, both locoregional and distant tumours appeared in 37.5, 50 and 12.5% as the first site of relapse in this study. Relapse patterns were analyzed in several studies previously. First relapse was noted in 11% of locoregional, 61% distant and 28% both local and distant in the tri-modal approach (Albain *et al*, 1995). In patients who received radiotherapy alone or concurrently with daily low dose cisplatin, the locoregional, distant and both relapse rates were 45, 36, 19% *vs* 38, 47, 14% respectively (Schaake-Koning *et al*, 1992). These observations suggest that both systemic and local treatment need to be improved in the future.

Patients with inoperable disease after chemotherapy, who did not respond to chemotherapy and who had delayed local treatment time had the worst prognosis in this series. Nine patients who did not reach operable criteria received concurrent chemoradiotherapy with daily low dose cisplatin. However, all nine patients in this series who received concurrent chemoradiotherapy as salvage treatment after they failed to respond to chemotherapy were progressed and eventually died. Patients who did not respond to induction chemotherapy may be inherently resistant to radiotherapy. A current EORTC study accruing larger number of patients using two cycles of GC plus radiotherapy with daily low dose cisplatin may be able to confirm this observation (Giaccone, 2000).

One patient in this series died of hepatitis B reactivation during induction chemotherapy. The hepatitis B virus carrier rate in Taiwan is around 15%. Hepatitis B reactivations were encountered frequently in hepatitis B virus carriers undergoing chemotherapy. Hepatitis B reactivation occurred in 20% of 78 carriers in a prospective study (Yeo *et al*, 2000). Two patients developed severe hepatitis B reactivation in 46 stage IIIB/IV NSCLC patients who received gemcitabine and oral etoposide (Mok *et al*, 2000). The use of steroid in chemotherapy may contribute to hepatitis B reaction. Since the natural histories of hepatitis B reaction in these patients are diverse, the relation between chemotherapy, steroid and hepatitis B reactivation are still not clear. The patient in this study received lamivudine after hepatitis B virus reactivation. However, this treatment was not always successful (Yeo *et al*, 1999).

In conclusion, three cycles of gemcitabine and cisplatin has demonstrated a good response rate in LAD-NSCLC patients. This regimen can be used in the induction treatment for LAD-NSCLC patients.
